# Violations of International Code of Breast‐milk Substitutes (BMS) in commercial settings and media in Bangladesh

**DOI:** 10.1111/mcn.13351

**Published:** 2022-03-21

**Authors:** Sifat P. Sheikh, Syeda M. Akter, Faugia I. Anne, Santhia Ireen, Jessica Escobar‐Alegria, Kirsten Kappos, Deborah Ash, Sabrina Rasheed

**Affiliations:** ^1^ Health Systems and Population Studies Division International Centre for Diarrhoeal Disease Research Bangladesh (icddr,b) Dhaka Bangladesh; ^2^ Division of Human Nutrition and Health Wageningen University and Research Wageningen The Netherlands; ^3^ FHI Solutions Washington District of Columbia USA

**Keywords:** Bangladesh, Bangladesh BMS Act, BMS Code, breast‐milk substitutes, breastfeeding, complementary feeding, food policy, infant and child nutrition, infant milk formula, low‐ and middle‐income countries, NetCode

## Abstract

The International Code of Marketing of Breast‐milk Substitutes (BMS) instituted to protect breastfeeding against unethical marketing, has been adopted by many countries, including Bangladesh. Despite national adoption, evidence suggests violations occur and inadequate BMS Code implementation is an issue. The study aimed to assess violations of the International BMS Code and the national ‘Breast‐milk Substitutes, Infant Foods, Commercially Manufactured Complementary Foods and the Accessories Thereof (Regulation of Marketing) Act, 2013’ of Bangladesh in commercial settings (retail outlets and media) in Bangladesh, for different types of milk, bottles, and teats using a standardized Network for Global Monitoring and Support for Implementation of the Code and Subsequent relevant World Health Assembly Resolutions (NetCode) protocol. This cross‐sectional quantitative study was conducted in Bangladesh from January to September 2018 in Dhaka, Chattogram, and Sylhet cities. Descriptive statistics were reported and *χ* ^2^ tests were conducted to assess differences between categorical variables of interest. Data were analysed using SPSS version 20. In retail outlets, there were higher proportion of violations observed in Dhaka than in Sylhet and Chattogram (*p* < 0.001). Significantly greater proportion of violations in product labels occurred among products sold without local distributors compared to others (*p* < 0.05); violations were higher among “other milk” for children aged 0 to <36 months compared to formulas and growing‐up milk (*p* < 0.05). Among media channels, internet clips had significantly higher proportions of violations compared to television, radio and newspaper (*p* < 0.001). BMS Code violations were prevalent in product labels and promotion of products through retail outlets. The study findings highlight the need for specific multisectoral strategies for better enforcement of BMS Code and points to the need for periodic assessment of Code violations.

## Key messages

- This is the first known study documenting violations in retail outlets, online stores, and media using the standard NetCode protocol in Bangladesh.
- BMS Code violations were high in retail stores and were more prevalent in the capital city compared to others.
- Violations were significantly higher in the internet compared to radio, television, and newspapers, highlighting the need for strengthening the monitoring of internet‐based promotions.
- It is essential to allocate resources to sensitize retail stores to the BMS Code to enhance the implementation of the law.

## Graphical abstract

In Bangladesh, BMS Code and Act violations were significantly higher in retail outlets of the capital city Dhaka compared to Sylhet and Chattogram cities; Displays were the most common type of promotion observed in large and small stores.

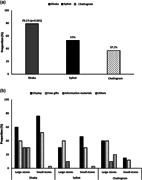

## 1 INTRODUCTION

Globally, it is estimated that optimal breastfeeding practices could help avert 823,000 deaths among under‐5 children and 20,000 maternal deaths annually (World Health Organization [WHO], 2021). Breastfeeding in early childhood can potentially prevent several infectious diseases, malnutrition and other noncommunicable diseases later in life (Baird et al., 2017). Despite the known benefits of breastfeeding, globally only 42% and in Bangladesh 65% of mothers exclusively breastfeed their infants for the first 6 months following birth (Gupta et al., 2019; National Institute of Population Research and Training and ICF International, 2019). In Bangladesh, 36% of females are employed, with a large number of them working in the readymade garments manufacturing industry (Haider & Thorley, 2020). As more women join the workforce (Rahman & Islam, 2013), mothers are facing challenges such as inadequate maternity leave, excessive workload, lack of skilled support for breastfeeding at health facilities and community, inadequate and low‐quality crèche facilities at work and unfavourable workplace policies (Hasan et al., 2020), which impede breastfeeding. Moreover, aggressive marketing of breast‐milk substitutes (BMS) play an important role in reducing the optimal duration of breastfeeding (Neves et al., 2021). Pervasive marketing of BMS have been shown to adversely impact knowledge, intention, beliefs, self‐efficacy and social norms related to breastfeeding (Green et al., 2021). COVID‐19 pandemic has created an additional threat to optimal breastfeeding, as BMS companies have capitalized on the fear of possible transmission of the infection through breast milk, to promote the use of BMS products (Ching et al., 2021). To counter this threat and maximize survival and health of newborn children globally and in Bangladesh, stakeholders must stay vigilant and act immediately to sustain the progress made at protecting, promoting and supporting breastfeeding.

The International Code of Marketing of BMS (hereinafter referred to as ‘the Code’) represents the international policy framework to protect breastfeeding against inappropriate and unethical marketing practices from manufacturers and distributors of BMS products (WHO, 1981; WHO & UNICEF, 2003). The Code was adopted by the World Health Assembly (WHA) in 1981 (Holla‐Bhar et al., 2015; WHO, 1981) and subsequently adopted by 139 countries including Bangladesh (Michaud‐Létourneau et al., 2019). In 1984, Bangladesh enacted the ‘Breast Milk Substitutes (Regulation of Marketing) Ordinance, 1984’ (Talukder et al., 2017; The Government of the People's Republic of Bangladesh, 1984), which was replaced by ‘The Breast‐milk Substitutes, Infant Foods, Commercially Manufactured Complementary Foods and the Accessories Thereof (Regulation of Marketing) Act, 2013’ (hereinafter referred to as ‘the Act’; The Government of the People's Republic of Bangladesh, 2013) and supported with additional bylaws in 2017. The Act included provisions that would allow it to supersede other conflicting laws or policies that are in existence (The Government of the People's Republic of Bangladesh, 2013). It also included guidelines and restrictions on importing BMS products, requirements for registering products, guidance on labelling and promotion of products through different outlets, and penalties for violations of the Code (The Government of the People's Republic of Bangladesh, 2013). To implement the Act, a national advisory committee was developed under the leadership of the Institute of Public Health Nutrition (IPHN) (Ministry of Health and Family Welfare, 2017). There are some differences between the Code and the Act. For instance, the age of the children included and guidance on violations among product labels and promotion in retail outlet stores (The Government of the People's Republic of Bangladesh, 2013). Where the Code and the Act align are with respect to regulations against inappropriate sales promotion of BMS through contents/messages in product labels, promotional activities in retail outlets, media content, and promotion of BMS at health facilities focusing on health care providers and mothers of young children (Michaud‐Létourneau et al., 2019; WHO, 2020). Despite nationwide adoption of BMS regulations, there has been significant challenges in implementation (Michaud‐Létourneau et al., 2019) and several violations of the Act were detected through a pilot project in Bangladesh (Bangladesh Breastfeeding Foundation, 2014). In previous studies conducted in Bangladesh, lack of knowledge of the Act and the absence of a strong monitoring and enforcement system were identified as reasons for violation of regulations by manufacturers, distributors and health professionals (WHO, 2020).

BMS Code violations in commercial settings such as retail outlets and media are considered crucial and researchers have reported existence of such violations from many countries around the world. In a recent study conducted in Ethiopia, researchers reported that majority of BMS Code violations happened through posters at retail outlets and television (TV) channels and all product labels violated at least one item of the Code (Laillou et al., 2021). A media analyses in Cambodia and Senegal revealed that BMS product advertisements claiming both health and nutritional benefits were frequently aired on local TV channels (Champeny et al., 2019). In Bangladesh, research reporting violations of BMS Code is scarce and have typically focused on health facilities (Taylor, 1998). However, there are a diverse range of marketing channels open to manufacturers and sellers of BMS products outside the health facility (Baker, Santos, et al., 2021). With growing numbers and types of retail outlets and media reach, it has become necessary to assess the violations of the Code in commercial settings and media in countries that adopted the Code (WHO, 2017b). In 2014, WHO and UNICEF established a Network for Global Monitoring and Support for Implementation of the Code and Subsequent relevant WHA Resolutions (NetCode) (WHO, 2017a). Through the network, specific methodologies were developed for monitoring and periodic assessment of BMS Code violations. NetCode recommends that periodic assessment is conducted every 3–5 years, to quantify the level of compliance with BMS Code (WHO, 2017a). In Bangladesh, the NetCode methodology has not been employed thus far. Our findings could be used to benchmark BMS Code violations in Bangladesh for future periodic assessments. The assessment of BMS Code violations will inform the design of targeted intervention to strengthen implementation, monitoring and enforcement of BMS Code in Bangladesh.

## 2 METHODS

### 2.1 Study design and settings

A cross‐sectional quantitative study aimed at assessing compliance with the Code and the Act in commercial settings, retail stores and media was conducted in Bangladesh from January to September 2018. We designed the study on the basis of the retail, label and media modules of NetCode toolkit protocol for periodic assessment (WHO, 2017a). The NetCode toolkit makes provision for monitoring and periodic assessment among key channels and primary respondents: mothers, health facilities, retail stores and media. The current analysis includes data from retail stores and media modules for BMS Code violations (Table 1). BMS products are any food or drink ‘being marketed or otherwise represented as a partial or total replacement for breast milk, whether or not suitable for that purpose’ and include any milk products, food, bottles and teats (Shubber, 2011). For our current analysis, we restricted our assessment to infant formula (0+ months), follow‐up or follow‐on formula (6+ months), growing‐up milk (12+ months), any other milk for children (0 to <36 months), bottles and teats (WHO, 2017a). We conducted a survey in the retail outlets of three major cities in Bangladesh: Dhaka, Chattogram and Sylhet. Evidence shows that large cities usually encompass the largest market share for any BMS product (Baker, Russ, et al., 2021; WHO, 2017a). Therefore, BMS product range available in shops and pharmacies in smaller cities and rural areas are not likely to exceed the product range available in the large cities. To select the retail outlets, we randomly selected two wards (administrative areas) of Dhaka and purposively selected one ward from each of Chattogram and Sylhet for the survey. For the media component of the study, a media firm conducted a retrospective scan of advertisements from all major local mass media channels (newspaper, radio and television) for a period of 6 months (January to June 2018) and data from the internet were collected prospectively for 2 months (August and September 2018), to capture the marketing and promotion of selected BMS products in Bangladesh. Promotions of BMS in the media are activities or dissemination of information for marketing, sales or distribution of the BMS products through media channels. The promotions could be through advertisement, information note, interview, information broadcasted through news reporting in TV/radio or newspaper, or internet (news report), viral internet marketing for specific product or brand, incentives for product promotion, sweepstakes, club membership, opinion, analysis, debate and others.

**Table 1:** Key channels for assessment of BMS Code violations in NetCode toolkit

Key channels/primary respondents	Included in current analysis	
Mothers	Mothers with at least one child <24 months of age	
Health facilities	Health professionals (public and private)	
Retail	Promotional activities and product labels in retail outlets	√
Media	TV channels, newspapers, radio, internet‐based advertisement	√

Abbreviations: BMS Code, International Code of Breast‐milk Substitute; NetCode, Network for Global Monitoring and Support for Implementation of the Code and Subsequent relevant WHA Resolutions; TV,  television.

### 2.2 The Code and the Act

We considered provisions from both the Code and the Act. There are differences between the two: the Code includes provision for children up to 36 months of age (WHO, 2017a), whereas the Act expanded the scope of restrictions to 0–5‐year‐old children (The Government of the People's Republic of Bangladesh, 2013; WHO, 2020). For product labels, the Act has a few requirements in addition to those described in the Code (Appendix A3). For product labels, the additional requirements for the Act were as follows: display of registration number from relevant authority in Bangladesh, size of the company logo should not be more than half of the size of the product name, must use words suitable or usable for a ‘child’ or other similar words, must use picture of baby or mother, or both, and have graphics or cartoon for easy identification of BMS. The Act prohibits any display of the BMS products in retail outlets (The Government of the People's Republic of Bangladesh, 2013). Therefore, this assessment reported a category named ‘any display’, which included those that do not fall within the scope of brand shelf, special display, shop window, poster, banners, shelf‐tags, talkers and product launch. For review of promotional materials collected from retail outlets, we added additional criteria related to the Act such as type of promotion found in the information notes (education information), price‐related promotions and free gifts, whether the promotional material contains information on the following: benefits of colostrum, initiation of breastfeeding within 1 h of birth, exclusive breastfeeding for 6 months, feeding homemade food in addition to breast milk after 6 months, importance of breastfeeding up to 2 years of age and information on appropriate way of breastfeeding and continuation thereof (The Government of the People's Republic of Bangladesh, 2013; WHO, 2020). In this study, we will report findings according to the Code, to enable transnational comparison and, whenever appropriate, we will include discussions about the Act.

### 2.3 Data collection

#### 2.3.1 Sample selection

To assess the labels of BMS products at retail outlets, we selected five large stores randomly through lottery from all the large stores mapped in Dhaka city as per NetCode methodology. Large stores were considered those selling a high volume and variety of BMS products (chain grocery stores, supermarkets, baby stores and chain pharmacies; Table 2). When one store refused (only occurred for two stores) to participate, it was replaced with another randomly selected store from the list. For assessment of the labels, we started in one large store, purchased a single item of every relevant product and took them to the central office to create a list and assess the product labels. All products found at the second store (not appearing on the existing list) was purchased and, accordingly, the list was updated. Similar procedure was followed for all five large stores until no new products were found. From large stores in Chattogram and Sylhet, we added a few additional BMS products that met the study criteria to our list for assessing product labels.

**Table 2:** Sample characteristics

	Number of stores sampled (total stores mapped)			
Characteristics	Dhaka	Sylhet	Chattogram	Total
I. Assessment of retail stores				
A. Large stores				
Grocery chain stores	2	2	2	6
Independent grocery stores/supermarkets	4	4	4	12
Chain independent (not associated with health facilities) pharmacies	2	2	2	6
Baby stores	2	2	2	6
Total large stores	43	43	43	129
B. Small stores				
Corner/convenience store	11	11	11	33
Small grocery (mudi dokan)/neighbourhood stores	11	11	11	33
Small independent (not associated with health facilities) pharmacies	11	11	11	33
C. Online stores				5
II. Assessment of media channels				
TV channels	6			
Radio channels	5			
Daily newspaper	8			
Internet clips	149			

Abbreviations: BMS, breast‐milk substitute; TV, television.

To assess in‐store promotion of BMS products, a two‐step process was performed to sample three types (large, small and online) of retail stores. Small stores included corner stores, stalls and neighbourhood stores or mudi dokan (small open‐fronted huts or cubicles) stocking a limited range of household goods and groceries, and non‐chain pharmacies that are not associated with any health facilities. First, we conducted physical mapping of all large and small stores around the selected areas in three cities by visiting the stores. In each city, 33 small stores and 10 large stores were sampled using a random number generator from the list of stores obtained through mapping. Data collected from a total of 129 stores in Dhaka, Sylhet, and Chattogram, were assessed for in‐store promotions (Table 2). For online stores, a list was made by searching the web and on the basis of local knowledge on online retailers that sell products within the scope of our study. We selected five online stores randomly from 18 online stores identified from web search. For the media assessment, we identified six major TV channels, five radio stations and eight daily newspapers (seven Bangla and one English) based on their popularity and reach.

#### 2.3.2 Data collection

For data collection at retail outlets, data collectors obtained permission from the store managers to take photos and conduct observation. All the unique products were purchased, photographed and given an identification number. The data from the labels were then summarized using a digital form that included company name and brand, product name, package size, recommended ages, product type, language used and criteria for both the Code and the Act. All types of promotions observed were photographed or a copy of promotional material was collected. Promotions included price‐related promotions (coupons, stamps, discounts and special discount sales), displays (brand shelves, special shop windows detached from a shelf, placards, posters, banners, shelf tags, talkers, wobblers, screamers and new product launches), promotion or education materials (posters, banners, coupon, flyers, leaflets, handbill, pamphlets or similar instruments), free gifts (to customers), product samples and the presence of company representatives in stores. For materials that were taken from the store (e.g., pamphlets, flyers and coupons), a matching label was fixed to the data collection form and the material. Paper‐based data collection forms were used for recording details of each promotion observed. For the online stores, we visited the websites of selected online stores. For every promotion encountered, a master list was created for all sites visited along with an electronic screenshot of each web page.

For the media scan, data on snapshots of newspaper, internet clips, audio and video clips were collected for review after removing duplicates and ensuring that they met the selection criteria. The data on the unique media clip was summarized with a prescribed form. The form recorded details on the type of promotion, source of promotion, brand, company, messages conveyed and type of products. All data collected were entered using online formats to reduce entry error.

#### 2.3.3 Data analysis

Data on product labels and promotional materials (including pictures and samples) from the retail outlets were reviewed and described using a separate prescribed form. Product labels data were analysed by using a predeveloped checklist that listed Code‐noncompliant answers to define ‘violation’. Additional criteria for the Act was added to the checklist. Promotional materials from retailers were also assessed using this form. The assessment identified brand, manufacturer, category, and frequency and type of promotions. Point‐of‐sale promotions were presented as the proportion of the stores promoting at least one product and also as the number of promotions and individual promotions in each site.

From the media scan, average number of advertisements per week (for TV, radio and newspaper) was calculated. The duration of advertisements (minutes/week) for TV and radio only was calculated by taking the sum of the durations (in seconds) of advertisements and dividing it by 60 and 26 (number of weeks in 6 months), to come up with mean duration of advertisement per week. From the media clips obtained, we assessed advertisements based on product category, brand, media source and type of messages (including promotion of breastfeeding) for all BMS products. Descriptive statistics were analysed and presented using Excel; *χ* ^2^ tests were performed using SPSS version 20 (IBM). To determine the level of significance, *p* < 0.05 was considered significant.

## 3 RESULTS

### 3.1 BMS Code violations in retail outlets

**Figure 1:**
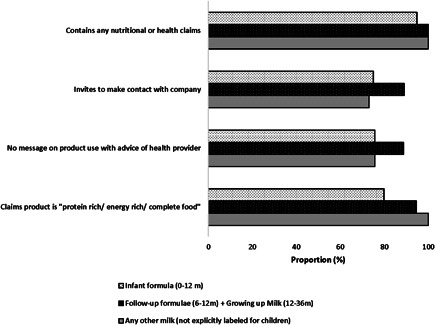
Categories of International Code of Breast‐milk Substitute (BMS) violations in product labels by type of BMS (milk) products

We identified a total of 106 unique BMS products of which 67 (63%) were milk products and the rest were bottles and teats. In terms of violation of the Act, almost all companies promoted their products as suitable for young children, the size of the logo was larger than the name of the product, did not contain statement about the superiority of breast milk and did not contain directions for preparing BMS product for consumption (Appendix A1). All the products identified violated one or more categories of the Code. There were some categories of the Code where the violation was quite high. Almost all (99%) of the milk products ‘made nutritional and health claims’ (Category 3 in Appendix A3) for their products, ‘invited the consumers to contact the company’ (Category 6 in Appendix A3), did not ‘contain language about consulting health care provider for starting BMS use’ (Category 26 in Appendix A3) and contained information about the ‘product being rich in different ingredients’ (Category 25 in Appendix A3 and Figure 1).

For labels of feeding bottles and teats (*n* = 39, 37% of BMS products), in terms of the Act, 94% of the products did not ‘contain word suitable or usable for a child or other similar words’ and 66% did not have the ‘registration number of Bangladesh printed on the label’ (Appendix A2). Major violations of the Code were observed in ‘use of language on the product label appropriate for Bangladesh’ (100%), lack of ‘batch number’ (94%), ‘pictures and texts idealizing bottles and teats, and by providing language about their suitability for children’ (87%) and lack of a ‘list of the ingredients’ they were made of (85%) (Appendix A2).

**Figure 2:**
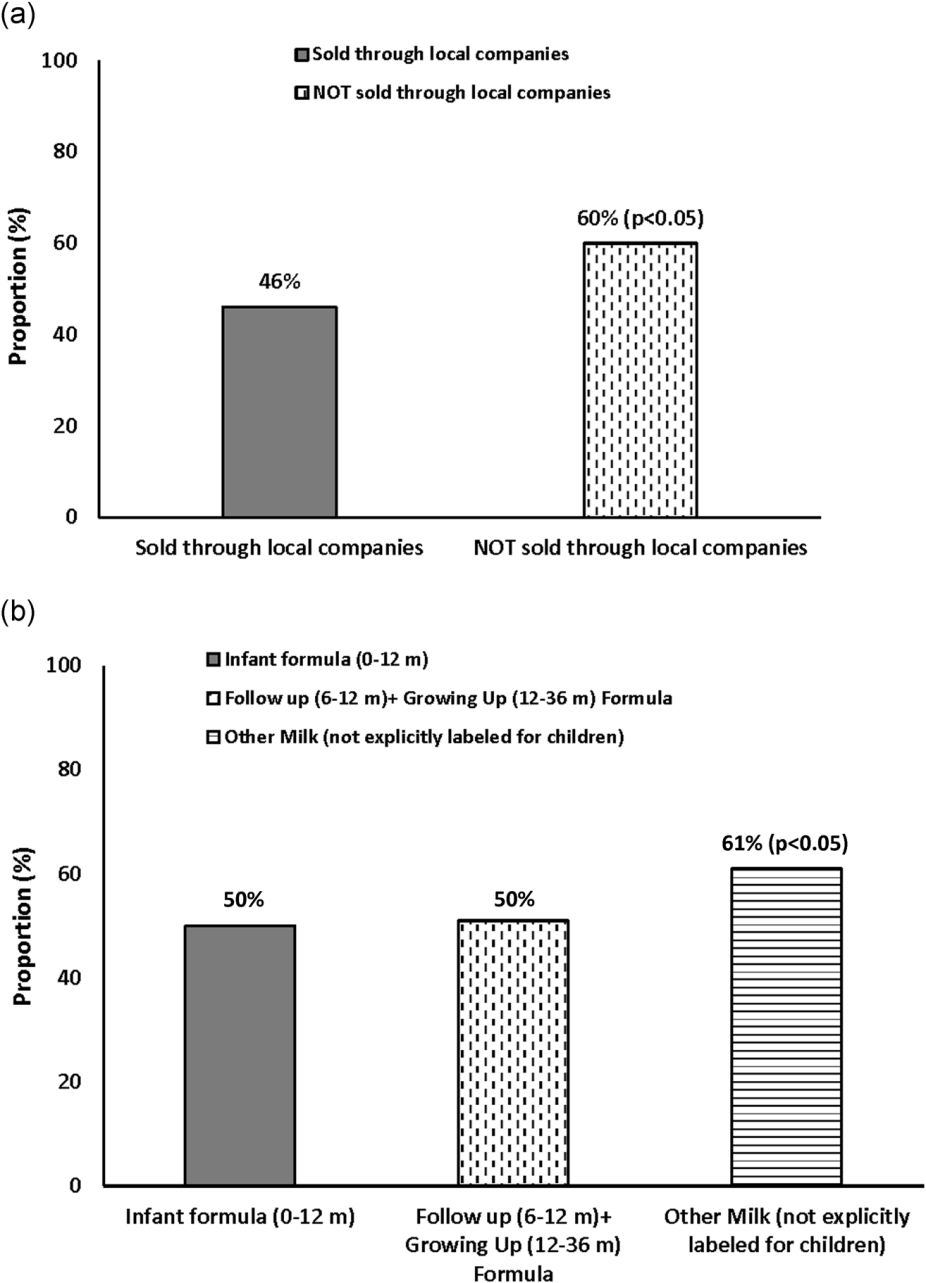
(a) Average proportion (%) of overall violations observed in breast‐milk substitute (BMS) milk products by type of companies. (b) Average proportion (%) of overall violations observed within different types of BMS milk products

Among the product labels, a third of the BMS milk products did not have any local company mentioned in the product label, whereas the rest of the labels mentioned a local company. The product labels that mentioned names of local companies as importers, distributers or as those involved in repackaging were categorized as ‘affiliated with a local company’ and those products that did not mention any local company on their labels were categorized as ‘not affiliated with a local company.’ Milk products affiliated with a local company had significantly lower proportion (46%) of violations overall, in their labels compared to those not affiliated with a local company (60%; *p* < 0.05). In terms of the type of BMS products, ‘other milk’ (61%) had significantly higher proportion of violations overall, in their labels, compared to the different types of formulas (50%; *p* < 0.05) (Figure 2).

**Figure 3:**
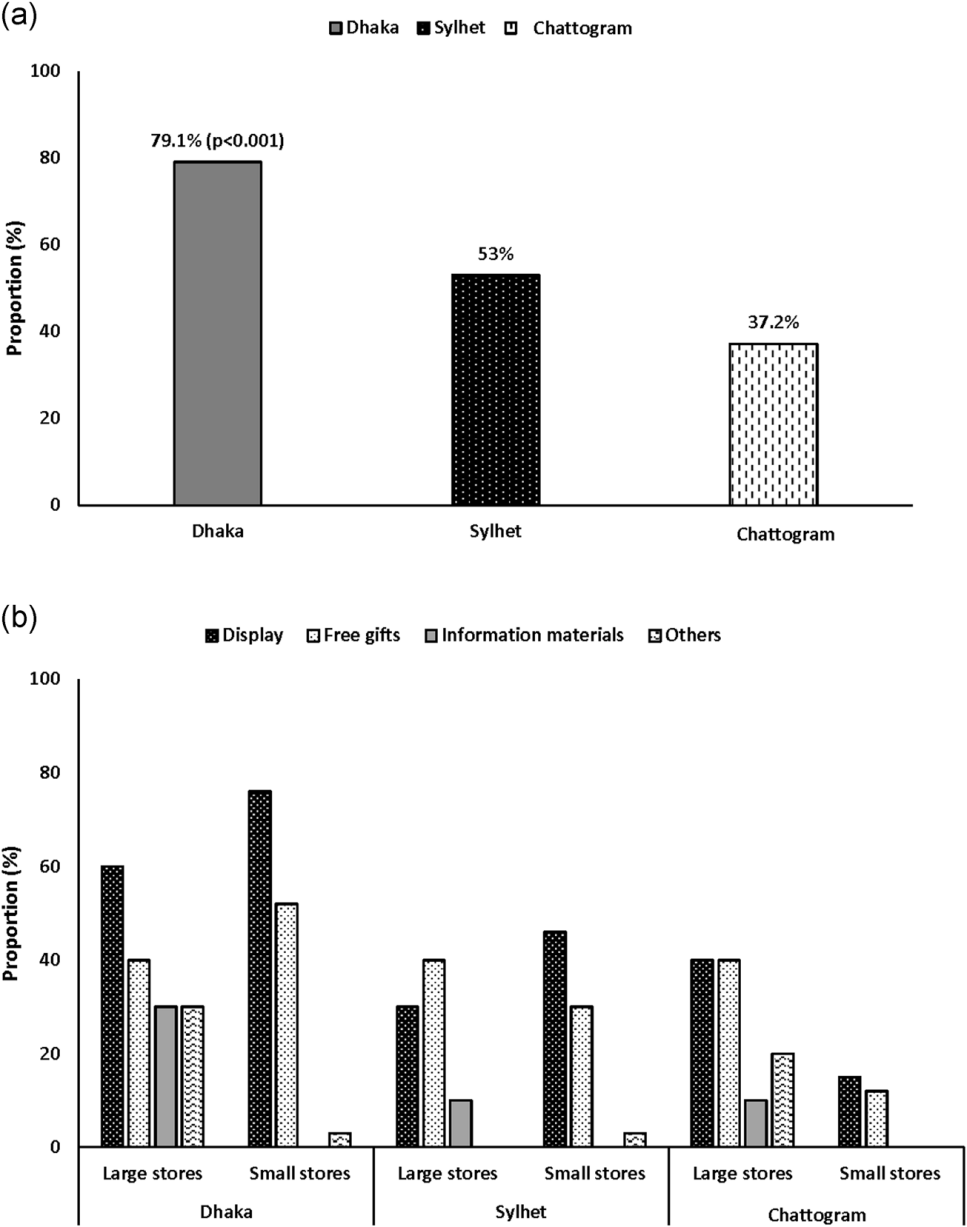
(a) Proportion (%) of any promotion observed in retail outlets by city. (b) Categories of promotions observed by types of stores and cities

A total of 945 promotions were observed in retail outlets. The Act did not permit any display of the BMS products. Based on this criterion, all the stores were in breach of the Act. When analysed based on the Code, 57% of all stores included in our sample had at least one violation. Promotion of BMS products (milk) was significantly higher in the capital city Dhaka followed by Sylhet and Chattogram (*p* < 0.001) (Figure 3). Display promotion and free gifts to customers were the major types of promotion observed in the stores assessed. In only one online store, price promotion for an infant formula was observed (data not displayed). In Dhaka and Sylhet cities, small stores such as corner stores, pharmacies and groceries were more likely to use display promotion for BMS products compared to the larger stores, although the difference was not statistically significant. However, in Chattogram city we observed that larger stores tend to have more promotions than the small stores, although the difference was not statistically significant (Figure 3).

### 3.2 BMS Code–violations in media

There were 243 unique clips identified during media scan that met our assessment criteria. Most of the unique clips were from internet (*n* = 154), followed by newspaper (*n* = 39), TV (*n* = 34) and radio (*n* = 16; Table 3). Advertisement was the most common promotion observed, followed by news reports and incentives for product purchase. In 31 clips, breastfeeding was promoted, which was observed on Mother's online forums only. In TV, radio and newspaper advertisements, violations were observed for ‘any other milk’ for children only (Table 3). We observed advertisements on significantly more types of BMS products on the internet compared to other media sources (*p* < 0.001) (Table 3). To advertise milk‐based BMS products, the claims were made about the product being nutritious, healthy and touting its contribution to child growth and building immunity. The feeding bottles were mostly advertised as convenient and healthy. The teats were advertised by focusing on its healthy composition, convenience of use and its similarity to mother's breast.

**Table 3:** Number and duration of advertisements by media source and type of products

Media source	No. of unique ads	No. of ads/week	No. of min/week
Radio (total)	16	256	80.5
Product type			
Infant formula		0	0
Follow‐up + growing‐up formulae		0	0
Any other milk for children		256	80.5
Feeding bottles and teats		0	0
Television (total)	34	195	65.7
Product type			
Infant formula		0	0
Follow‐up + growing‐up formulae		0	0
Any other milk for children		195	65.7
Feeding bottles and teats		0	0
Newspaper (total)	39	3.4	
Product type			
Infant formula		0	
Follow‐up + growing‐up formulae		0	
Other milk		3.4	
Feeding bottles and teats		0	
Internet promotion by manufacturer^a^ (total)	154		
Product type			
Infant formula			
Follow‐up + growing‐up formulae	9		
Other milk	97		
Feeding bottles	37		
Teats	29		

^a^ More than one type of product was promoted through multiple clips.

## 4 DISCUSSION

The findings of the current study reveal considerably high‐levels of violations of BMS Code in the commercial settings of retail outlets and in the media, especially the internet, in Bangladesh. In terms of promotion of BMS in retail outlets, proportion and type of violations differed by city. Product label violations were universal and higher in products that did not have local company involved in distribution. The traditional media such as radio, TV and newspaper were quite compliant to BMS Code. However, a wide range of BMS products were promoted through social media and webpages. Although violations of BMS Code has been reported from Bangladesh previously (Joshi et al., 2014; Rahman & Akter, 2019; Taylor, 1998), this is the first study documenting violations in retail stores, online stores and media using the standard NetCode protocol (WHO, 2017a).

We found that all the labels of BMS products violated at least one aspect of both the Act and the Code. In studies conducted in Turkey, Cambodia and Mexico comparably high levels of the Code violation in product labels were reported by researchers (Ergin et al., 2013; Hernández‐Cordero et al., 2019; Hou et al., 2019). The level of violations in product label calls for serious attention to the existing policy and monitoring mechanism around BMS Code violation. Further, market leaders among BMS companies should be engaged to find a way to improve compliance to the Code. We found that BMS products associated with local companies were more likely to comply with BMS Code compared to products that are not directly linked to local companies. This is probably because local companies are more aware of the Act and, therefore, tried to comply with the local regulations. However, quite a number of BMS products were sourced without linkage to local companies. Despite existing rules about BMS import, it is crucial to understand how to adequately monitor the channels of BMS import so that compliance to the Act can be ensured. It is also important to understand the existing mechanism that monitor the retail stores so that products that do not comply with the BMS Code and Act are not sold to the consumers.

We observed a high proportion of promotion of BMS products in the different retail outlets. The highest proportion of BMS Code violations occurred in the retail stores of Dhaka city compared to the other two cities. This is expected as Dhaka is the capital and the largest city in Bangladesh and probably has the largest share of the BMS market. NetCode protocol, also specified that for in‐country assessment of BMS Code violation, the data should be collected from the largest city in the country (WHO, 2017a). However, it is important to note that the pattern of promotions of BMS products varies among cities and this information could be useful to formulate city specific enforcement strategies in the future. Further the pattern of promotions of BMS products differed by store type and should be kept in mind for development of future activities related to sensitization of managers and staff, and monitoring of retail outlets. According to the Act, any type of display of BMS products are considered a violation. It is important to evaluate the feasibility of such a provision for retail outlets. Whether such stringent regulations are feasible in the context of Bangladesh and setting more realistic expectations on the display of BMS products in retail stores is a matter worth revisiting by policymakers.

In the media outlets, we found very few advertisements of BMS products, which shows that there have been efforts to comply with BMS Code in the major mainstay media channels such as television, radio and newspaper. The few advertisements for milk products observed were of ‘other milk’, which were not specifically labelled for any specific age group, particularly for infants or young children. Nevertheless, these products were advertised as ‘nutritious’, ‘healthy’, and ‘good for growth and development of the children’, implying that the products might be consumed by young children. Similar findings from media audit reported inappropriate promotion of milk products (not just other milk) in other countries of Southeast Asia (Vinje et al., 2017). One important issue to consider is that the Act includes under‐5 children and ‘other milk’ is an important source of nutrition for young children. Therefore, for the Act it is important to come up with guidance about ‘other milk’ that protects breastfeeding but promotes the use of milk for older children. We found most media promotions of BMS products were conducted through internet. It is important that newer media channels such as internet are monitored by relevant government authority for BMS Code violations and necessary steps taken to strengthen enforcement.

In Bangladesh, the rates of exclusive breastfeeding have stagnated in recent years (Hossain et al., 2018; Rahman et al., 2020) and use of BMS products have risen (Rollins et al., 2016). In this situation, it is crucial that BMS Code violations in commercial sectors are adequately dealt with so that a supportive environment for breastfeeding for mothers can be ensured in Bangladesh. It was very clear from our study that the enforcement of the BMS Code need to happen with active involvement of multiple sectors within and outside the government. During the World Breastfeeding Week 2021, Bangladesh ranked first in the world, based on infant and young child feeding indicators on the World Breastfeeding Trends Initiative (WBTi) criteria (WBti, 2021). However, WBTi assessment based on national level adoption of regulation and supportive policy environment, did not assess enforcement of Act using the actual levels of violation that exists in the country. To ensure enforcement of strong legislations such BMS Code, it is important to strengthen monitoring efforts and conduct periodic country assessment using NetCode protocol. The conflict between commercial interest and public health is not a new phenomenon. However, to promote and protect breastfeeding—a lifesaving measure for infant and young children from commercial influence—BMS Code is a crucial policy instrument. It is essential, therefore, that resources are allocated to sensitize people about the BMS Code and create mechanisms to reduce Code violation in Bangladesh and other countries.

Our study had a number of limitations. We only looked at milk products, bottles and teats while there are other categories of BMS products (e.g., complementary foods), which are being sold but were not considered due to resource constraint. It must be noted that there are several companies that use marketing strategies to promote BMS products as complementary foods (Berry et al., 2010). We assessed stores located in three major city corporations of Bangladesh and, therefore, our findings may not be applicable for smaller cities or rural areas. For media analysis, we considered local media sources but did not include international TV channels that are pervasive. The analyses of internet marketing were limited to a few publicly available sites and pages, whereas there may be other social media pages or sites that allow moderator‐regulated or consumer‐targeted access that we missed out.

There are two noticeable strengths of the study. First, the study was undertaken under the leadership and technical guidance of the IPHN of the Ministry of Health and Family Welfare, which is the government agency to implement and monitor that Act. This ensured government ownership of the findings from outset. Second, the study used the NetCode toolkit. The research team successfully contextualized and adapted the global recommendations on standard methods and tools. The current study was set to successfully contextualize WHO globally recommended research methods for obtaining locally and globally relevant results for advocacy and collaborations to advance the Code compliance (led by GoB) through policies and programmes that promote breastfeeding. Periodic assessment will be important to understand the trends in the Code violation and this information will be valuable for future policy and programme formulation.

## 5 CONCLUSIONS

This study highlights that violations of the BMS–Code in commercial settings is highly prevalent in Bangladesh. A multisectoral approach involving policymakers, BMS industry leaders, distributers, shopkeepers, media representatives and consumers is necessary to control Code violations across the country. It is important that ambiguities between the Code and the Act are–assessed and monitoring tools are developed to enhance compliance. Efforts should be made to create awareness about the importance of the registration of the BMS products for the consumers and retail outlets so that there is an increased awareness and reporting of violation of BMS regulations in Bangladesh.

## CONFLICTS OF INTEREST

The authors declare no conflicts of interest.

## ETHICS STATEMENT

Local ethical clearance was obtained from the Ethical Review Committee of icddr,b before conducting the survey of retail outlets (icddrb protocol #: PR‐18022). The Institutional Review Board of FHI 360 acknowledged the study as exempt (Project #: 1245006‐3). Written informed consent was obtained from all subjects involved in the study. The secondary analysis of the data from media scan was exempt from review.

## AUTHOR CONTRIBUTIONS

Sabrina Rasheed, Santhia Ireen, Kirsten Kappos and Jessica Escobar‐Alegria contributed to the conception and design of the study. Sabrina Rasheed, Sifat P. Sheikh, Syeda M. Akter and Faugia I. Anne contributed to data acquisition. Santhia Ireen, Sabrina Rasheed, Kirsten Kappos, Jessica Escobar‐Alegria and Deborah Ash acquired funding for this project. Sifat P. Sheikh and Syeda M. Akter contributed to data analysis and interpretation. Sifat P. Sheikh and Sabrina Rasheed drafted the manuscript. Santhia Ireen, Jessica Escobar‐Alegria, Kirsten Kappos and Deborah Ash reviewed and made technical contribution in the document. All authors critically revised the manuscript, agree to be fully accountable for ensuring the integrity and accuracy of the work, and read and approved the final manuscript.

## Data Availability

The data we used in this manuscript are available upon request to researchers via Armana Ahmed (armana@icddrb.org).

## 1

See Tables A1, A2, and A3

**Table A1:** Number of violations of BMS code by type of milk products (Bangladesh Act)

Category	Description	Infant formula (*n* = 20)	Follow‐up + growing‐up formulae (*n* = 36)	Any other milk (*n* = 15)
3	Contains any nutritional or health claims	19	36	15
6	Includes invitation to make contact (direct/indirect with the company)	15	32	11
15^a^	Contains word suitable or usable for a child or other similar words	20	36	15
17	Contains the statement on the superiority of breast milk	20	32	15
18^a^	Size of the logo is not more than half of the size of the name of the commodity	18	35	14
21^a^	Contains texts or images that may idealize the use of breast milk substitutes	17	34	13
24	Contains a statement that the products should be used only on the advice of the health worker/Contains the message that 'the product is approved/advised by physicians' or similar information	17	32	11
25	Contains the information such as that 'the product is protein rich' or 'energy rich', or 'complete food'	16	34	15
28	Contains the warning message 'Pathogenic microorganisms can be present in breast milk substitutes. It should be prepared following the standard procedure'	17	25	12
30^a^	Contains the rules of use/instruction guide/prescribed description	18	33	14

Abbreviation: BMS, breast‐milk substitute.

^a^ Bangladesh Code only.

**Table A2:** Proportion (%) of violation in labels of feeding bottles and teats

Category	Description of categories applicable for bottles and teats	Bottles and teats (*N* = 35)	
		*n*	Violation %
1	Product information is printed on the container or well attached label	0	–
2	The language used on the product label is appropriate for Bangladesh	35	100
3	Contains any nutritional or health claims	20	61
4	Includes the recommended appropriate age of introduction	8	23
5	Conveys an endorsement by a health worker or health professional body	0	–
6	Includes invitation to make contact (direct/indirect with the company)	20	61
7	Contains promotional devices to induce sales of the company's products under the scope	4	12
8	Includes list of ingredients	28	85
10	Contains storage instruction	4	12
11	Batch number printed on the label	31	94
12	Date of expiry printed on the label	24	73
13^a^	Date of manufacture printed on the label^a^	22	67
14^a^	Registration number of Bangladesh printed on the label^a^	23	66
15^a^	Contains word suitable or usable for a child or other similar words^a^	31	94
31	Contains images or texts that idealize the use of feeding bottles and teats	31	87

^a^ Bangladesh Code only.

**Table A3:** Description of categories of BMS National Act

Category	Description of categories
1	Product information is printed on the container or well attached label
2	The language used on the product label is appropriate for Bangladesh
3	Contains any nutritional or health claims
4	Includes the recommended appropriate age of introduction
5	Conveys an endorsement by a health worker or health professional body
6	Includes invitation to make contact (direct/indirect with the company)
7	Contains promotional devices to induce sales of the company's products under the scope
8	Includes list of ingredients
9	Displays nutritional composition of the product
10	Contains storage instruction
11	Batch number printed on the label
12	Date of expiry printed on the label
13	Date of manufacture printed on the label
14^a^	Registration number of Bangladesh printed on the label
15^a^	Contains word suitable or usable for a child or other similar words
16	Includes the words 'important Notice' or their equivalent
17	Contains the statement on the superiority of breast milk
18^a^	Size of the logo is not more than half of the size of the name of the commodity
19^a^	Contains picture of baby or mother or both
20^a^	Contains graphics or cartoon for easy identification of breast milk substitute
21	Contains texts or images that may idealize the use of breast milk substitutes
22	Contains text or images that may discourage or undermine breastfeeding
23	Contains information that implies a belief that BMS products are equivalent or superior to BM
24	Contains a statement that the products should be used only on the advice of the health worker/Contains the message that 'the product is approved/advised by physicians' or similar information
25	Contains the information such as that 'the product is protein rich' or 'energy rich,' or 'complete food'
26	Contains a statement on the need for health worker's advice on the proper method of use
27	Contains a warning against the health hazards of inappropriate preparation and uses
28	Contains the warning message 'Pathogenic microorganisms can be present in breast milk substitutes. It should be prepared following the standard procedure'
29	Contains the warning message 'breast milk substitutes, infant foods not the real source for nutrition'
30	Contains the rules of use/instruction guide/prescribed description
31	Contains images or texts that idealize the use of feeding bottles and teats (for bottles and teats only)

Abbreviation: BMS, breast‐milk substitute.

^a^ Bangladesh code only.
